# Supplementary material for: A Novel Co-Crystal of Bexarotene and Ligustrazine Improves Pharmacokinetics and Tissue Distribution of Bexarotene in SD Rats
Source: Pharmaceutics. 2020 Sep 23;12(10):906. doi: 10.3390/pharmaceutics12100906 (PMC7598278; doi:10.3390/pharmaceutics12100906)

checkCIF/PLATON report

Structure factors have been supplied for datablock(s) 2BEX-LIG

THIS REPORT IS FOR GUIDANCE ONLY. IF USED AS PART OF A REVIEW PROCEDURE FOR PUBLICATION, IT SHOULD NOT REPLACE THE EXPERTISE OF AN EXPERIENCED CRYSTALLOGRAPHIC REFEREE.

No syntax errors found. CIF dictionary Interpreting this report

Datablock: 2BEX-LIG

```
Bond precision:  C-C = 0.0030 A         Wavelength=1.54178

Cell:            a=49.28(2)     b=8.517(3)          c=11.787(4)
                 alpha=90       beta=100.410(5)     gamma=90
Temperature:     566 K

                Calculated                 Reported
Volume          4866(3)                    4866(3)
Space group     C 2/c                      C 1 2/c 1
Hall group      -C 2yc                     -C 2yc
Moiety formula  C24 H28 O2, 0.5(C8 H12 N2) C24 H28 O2, C4 H6 N
Sum formula     C28 H34 N O2               C28 H34 N O2
Mr              416.56                     416.56
Dx,g cm-3       1.137                      1.137
Z               8                          8
Mu (mm-1)       0.546                      0.546
F000            1800.0                     1800.0
F000'           1804.85
h,k,lmax        60,10,14                   60,10,14
Nref            4842                       4729
Tmin,Tmax       0.897,0.897
Tmin'           0.897

Correction method= Not given

Data completeness= 0.977          Theta(max)= 72.679

R(reflections)= 0.0621( 2886)     wR2(reflections)= 0.1887( 4729)

S = 0.961                  Npar= 296
```

The following ALERTS were generated. Each ALERT has the format
test-name_ALERT_alert-type_alert-level.
Click on the hyperlinks for more details of the test.

Alert level C

```
PLAT230_ALERT_2_C Hirshfeld Test Diff for    C18      --C19      .        6.0 s.u.
PLAT230_ALERT_2_C Hirshfeld Test Diff for    C19      --C21      .        6.0 s.u.
PLAT241_ALERT_2_C High   'MainMol' Ueq as Compared to Neighbors of        C18 Check
PLAT242_ALERT_2_C Low    'MainMol' Ueq as Compared to Neighbors of        C16 Check
PLAT242_ALERT_2_C Low    'MainMol' Ueq as Compared to Neighbors of        C19 Check
PLAT906_ALERT_3_C Large K Value in the Analysis of Variance ......      5.214 Check
PLAT911_ALERT_3_C Missing FCF Refl Between Thmin & STh/L=    0.600         17 Report
```

Alert level G

```
PLAT003_ALERT_2_G Number of Uiso or Uij Restrained non-H Atoms ...          3 Report
PLAT007_ALERT_5_G Number of Unrefined Donor-H Atoms .............           1 Report
PLAT042_ALERT_1_G Calc. and Reported MoietyFormula Strings  Differ     Please Check
PLAT072_ALERT_2_G SHELXL First  Parameter in WGHT  Unusually Large       0.11 Report
PLAT128_ALERT_4_G Alternate Setting for Input Space Group     C2/c       I2/a Note
PLAT177_ALERT_4_G The CIF-Embedded .res File Contains DELU Records          2 Report
PLAT432_ALERT_2_G Short Inter X...Y Contact  O1       ..C27              3.02 Ang.
                                                x,-y,-1/2+z  =      6_555 Check
PLAT860_ALERT_3_G Number of Least-Squares Restraints ............           2 Note
PLAT910_ALERT_3_G Missing # of FCF Reflection(s) Below Theta(Min).          2 Note
PLAT912_ALERT_4_G Missing # of FCF Reflections Above STh/L=  0.600         95 Note
PLAT933_ALERT_2_G Number of OMIT Records in Embedded .res File ...          8 Note
PLAT941_ALERT_3_G Average HKL Measurement Multiplicity ..........         3.8 Low
PLAT978_ALERT_2_G Number C-C Bonds with Positive Residual Density.          1 Info
```

---

0 **ALERT level A** = Most likely a serious problem - resolve or explain
0 **ALERT level B** = A potentially serious problem, consider carefully
7 **ALERT level C** = Check. Ensure it is not caused by an omission or oversight
13 **ALERT level G** = General information/check it is not something unexpected

1 ALERT type 1 CIF construction/syntax error, inconsistent or missing data
10 ALERT type 2 Indicator that the structure model may be wrong or deficient
5 ALERT type 3 Indicator that the structure quality may be low
3 ALERT type 4 Improvement, methodology, query or suggestion
1 ALERT type 5 Informative message, check

It is advisable to attempt to resolve as many as possible of the alerts in all categories. Often the minor alerts point to easily fixed oversights, errors and omissions in your CIF or refinement strategy, so attention to these fine details can be worthwhile. In order to resolve some of the more serious problems it may be necessary to carry out additional measurements or structure refinements. However, the purpose of your study may justify the reported deviations and the more serious of these should normally be commented upon in the discussion or experimental section of a paper or in the "special_details" fields of the CIF. checkCIF was carefully designed to identify outliers and unusual parameters, but every test has its limitations and alerts that are not important in a particular case may appear. Conversely, the absence of alerts does not guarantee there are no aspects of the results needing attention. It is up to the individual to critically assess their own results and, if necessary, seek expert advice.

Publication of your CIF in IUCr journals

A basic structural check has been run on your CIF. These basic checks will be run on all CIFs submitted for publication in IUCr journals (*Acta Crystallographica*, *Journal of Applied Crystallography*, *Journal of Synchrotron Radiation*); however, if you intend to submit to *Acta Crystallographica Section C* or *E* or *IUCrData*, you should make sure that full publication checks are run on the final version of your CIF prior to submission.

Publication of your CIF in other journals

Please refer to the *Notes for Authors* of the relevant journal for any special instructions relating to CIF submission.

PLATON version of 10/08/2020; check.def file version of 06/08/2020

Datablock 2BEX-LIG - ellipsoid plot

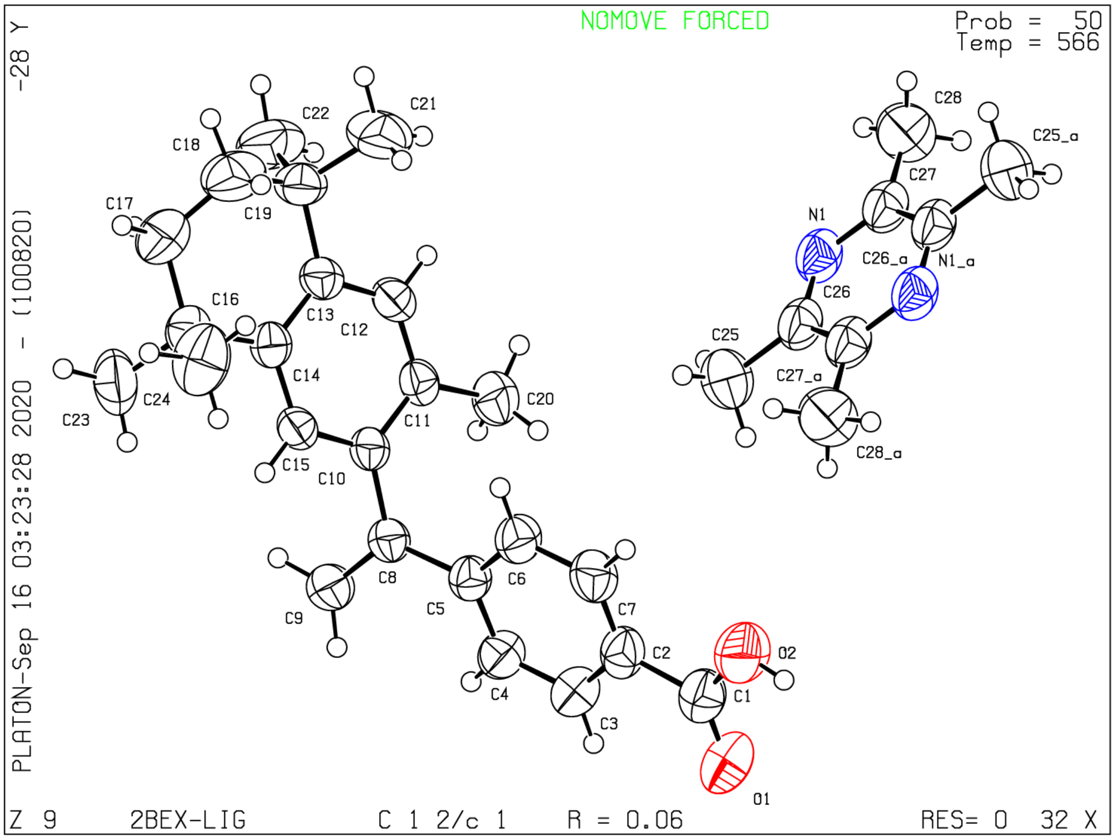

Supplement: Supplementary file 1 [file pharmaceutics-12-00906-s001.zip › supplement/checkcif.pdf]
